# Effects of Resistant Starch on Metabolic Markers and Gut Microbiota in Women with Metabolic Syndrome Risk Factors: A Randomized, Double-Blind, Pilot Study

**DOI:** 10.3390/nu17233652

**Published:** 2025-11-21

**Authors:** Kyu-Nam Kim, Min-Sook Kang, Nam-Seok Joo, Hyang-Rae Lee, Susie Jung, Seyoung Ju, Yong-Ju Lee, Kyucheol Lee, Soohwan Jung, Jisoon Im, Jiyoung Lee

**Affiliations:** 1 Department of Family Practice and Community Health, Ajou University School of Medicine, Suwon 16499, Republic of Korea; 2 Department of Biochemistry & Molecular Medicine, GW Cancer Center, School of Medicine & Health Sciences, The George Washington University, Washington, DC 20037, USA; 3 Department of Food Sciences, National Institute of Crop and Food Sciences, Rural Development Administration, 181, Hyeoksin-ro, Iseo-myeon, Wanju-Gun 55365, Republic of Korea; 4 Department of Food & Nutrition, College of Biomedical and Health Science, Konkuk University, Glocal Campus, Chungju 27478, Republic of Korea; 5 Cheongdamrui Plastic Surgery, Jeju 63083, Republic of Korea; 6 Refrery Sarang Clinic, Suwon 16500, Republic of Korea

**Keywords:** resistant starch, metabolic syndrome, gut microbiota, women

## Abstract

**Background/Objectives:** Resistant starch intake has been shown to influence gut microbiota composition and affect metabolic markers. These effects may be partially attributed to enhanced short-chain fatty acid (SCFA)-mediated energy harvesting and hepatic lipogenesis induced by resistant starch fermentation. However, there is a lack of prospective research addressing these associations. To address this gap, we performed a double-blind, randomized dietary intervention study to investigate the impact of high versus low resistant starch consumption on metabolic markers and gut microbiota among adult women presenting with risk factors for metabolic syndrome. **Methods:** A total of 30 participants were randomly assigned to either the low-resistant starch (LRS) or high-resistant starch (HRS) diet groups. Each group, comprising 15 participants, consumed one food product per day enriched with either high or low resistant starch for 8 weeks. Changes in metabolic indices and gut microbiota were assessed and compared with baseline values, as assessed before diet (Week 0). **Results:** After 8 weeks of intervention, the HRS diet significantly increased body weight, body fat, and triglyceride (TG) level (mean change ≈ +40 mg/dL), while reducing blood pressure. Analysis of intestinal microbiota in the HRS group revealed a statistically significant increase in the genus *Veillonella* following the intervention. Conversely, the genus *Marvinbryantia* increased significantly in the LRS group. **Conclusions:** In women with metabolic risk factors, resistant starch supplementation elicited mixed metabolic responses—showing a modest reduction in blood pressure but concurrent increases in adiposity and TG concentrations. As the TG elevation reached a clinically meaningful magnitude, dietary interventions involving high-resistant starch should incorporate regular lipid monitoring to ensure cardiometabolic safety. Collectively, these findings highlight the complex interplay between SCFA-producing gut microbiota and host energy metabolism, suggesting that individualized dietary strategies may be required to optimize metabolic outcomes.

## 1. Introduction

Carbohydrates are broadly classified into simple sugars—including monosaccharides and disaccharides—and complex carbohydrates, which encompass oligosaccharides and polysaccharides. Complex carbohydrates generally offer greater health benefits compared to simple sugars [1,2]. Within polysaccharides, dietary fiber is subdivided into soluble and insoluble categories. Soluble dietary fibers, such as gums, pectins, and β-glucans, can form viscous gels in the gastrointestinal tract, resulting in delayed glucose absorption and regulation of postprandial glycemia [3]. Conversely, insoluble dietary fibers, including cellulose, hemicellulose, and chitin, do not dissolve in water and transit the digestive tract with minimal alteration, enhancing stool bulk and supporting bowel regularity [4]. Resistant starch (RS) is classified as an insoluble dietary fiber that escapes digestion in the small intestine and moves to the colon, where it functions as a fermentable substrate for the gut microbiota [5]. RS exists in several distinct forms (RS1–RS5), each differing in its structural characteristics and formation mechanisms. Naturally occurring types (RS1 and RS2) are present in plant-based foods such as unripe bananas, legumes, and whole grains, whereas other types (RS3–RS5) are generated during food processing through starch retrogradation, chemical modification, or complex formation with lipids [6,7,8]. These RS variations affect the physicochemical properties and physiological effects, contributing to its diverse metabolic roles [6,7,8]. Fermentation of RS produces short-chain fatty acids (SCFAs), which participate in shaping gut microbiota composition and diversity, maintain intestinal biology, and may support improved metabolic outcomes [5,9,10]. Beyond these prebiotic functions, RS is linked to enhanced glycemic regulation and favorable changes in lipid metabolism, such as decreased blood glucose, triglycerides (TG), and cholesterol levels [9,10,11].

The metabolic syndrome refers to a set of metabolic disturbances—including central obesity, increased fasting blood glucose and TG levels, lowered high-density lipoprotein (HDL) cholesterol, and raised blood pressure—that considerably heighten the risk of type 2 diabetes and cardiovascular disease [12]. In South Korea, the proportion of individuals with metabolic syndrome now exceeds 30%, highlighting a serious public health issue [13]. The concurrent escalation in healthcare costs and socioeconomic impact underscores the critical importance of preventive efforts that emphasize diet and lifestyle modifications.

Despite the known health-promoting properties of RS, consumption levels among the Korean population remain comparatively low relative to those in other countries [14]. Although previous randomized controlled trials have primarily focused on the effects of RS on metabolic parameters in individuals with metabolic syndrome [15,16,17], limited research has examined its concurrent influence on gut microbiota composition and function. Therefore, our study aimed to investigate whether low- and high-resistant starch (LRS and HRS) intake affect metabolic biomarkers and gut microbiota profiles when an RS-enriched food formulation is administered once daily to women at risk of metabolic syndrome.

## 2. Materials and Methods

### 2.1. Study Participants

To minimize age-related variability and ensure sample homogeneity, women aged 40 years or older—including both premenopausal and postmenopausal individuals—were enrolled in the study. Because no prior prospective data were available to support a formal sample size calculation, this investigation was conducted as a preliminary pilot study from March to September 2024. A pragmatic sample of 15 participants per group was selected (based on commonly accepted pilot-study guidelines and to allow for potential attrition) [18], with the goal of estimating outcome variability and informing sample size calculations for a future definitive trial. Eligibility required the presence of at least two of the following criteria related to metabolic health: (1) waist circumference ≥ 85 cm, (2) fasting blood glucose ≥ 100 mg/dL, (3) TG ≥ 150 mg/dL, (4) HDL cholesterol ≤ 50 mg/dL, and (5) blood pressure ≥ 130/85 mmHg [19]. Exclusion criteria included medication use for inflammatory bowel disease or other gastrointestinal conditions, uncontrolled hypertension or diabetes mellitus, thyroid disorders or Cushing’s syndrome, current use of steroids, antibiotics, or probiotics, and ongoing treatment for depression, anxiety, or sleep disorders. Eligible participants were randomly allocated in a 1:1 ratio to either the HRS group (*n* = 15) or the LRS group (*n* = 15). Randomization was managed using independent codes generated and held by a coordinator not involved in the intervention at the dietary study center. Both participants and research staff remained blinded to group assignments, maintaining a double-blind protocol. The detailed composition of LRS and HRS is presented in Table 1, with both substitutes matched for caloric content and daily total energy intake between groups. The RS content of all test products (mixed-grain powder, cereal, and energy bar) was quantified by the National Institute of Crop and Food Science, Rural Development Administration (RDA, Republic of Korea), using the AOAC Official Method 2002.02 and the Megazyme Resistant Starch Assay Kit (K-RSTAR, Megazyme Ltd., Bray, Ireland). Written informed consent was obtained from all participants before study enrollment. The study was conducted in compliance with the Declaration of Helsinki. The protocol was reviewed and approved by the Ajou University Hospital Ethics Committee (AJIRB-MEDFOD-20-064).

### 2.2. Intervention

Participants were provided with an 8-week supply of test products, which were developed by the Rural Development Administration and delivered to their homes weekly. The test products, available in both cereal and energy bar formats, were designed for convenient consumption. Study instructions directed participants to consume the product once daily, five days per week, as a substitute for breakfast. If breakfast consumption was not feasible, the product was to be consumed as a snack between meals.

Following the acquisition of informed consent and a basic overview of the study, participants underwent baseline assessments, which included a body composition analysis (using InBody 720, Inbody Ltd., Seoul, Republic of Korea) and blood sample collection. Additional measurements encompassed fecal calprotectin analysis (Green Cross Labs, Republic of Korea) and gut microbiome profiling (CJ Bioscience, Seoul, Republic of Korea) using stool specimens. Participants were instructed to collect stool samples at home and bring them to the follow-up visit, and to submit a 3-day food diary each week (comprising 2 weekdays and 1 weekend day). They were also encouraged to engage in light physical activity about five times per week and to document the type and duration (in minutes) of each session in a physical activity diary. Approximately four weeks post-randomization, participants were contacted via telephone to monitor adherence to the intervention protocol (e.g., start date and compliance) and were reminded to maintain their food diary. In addition to diary and telephone monitoring, adherence was confirmed by monthly return counts of remaining test products. At the end of the 8-week intervention, participants attended a final visit during which all baseline assessments were repeated, and they were directed to submit the food and exercise logs from the last week.

### 2.3. Primary and Secondary Endpoints

This study was designed as an exploratory pilot trial to assess whether RS intake could improve metabolic risk factors in women exhibiting at least two components of metabolic syndrome. The primary endpoint was defined as the change in metabolic syndrome–related parameters, including systolic and diastolic blood pressure, fasting glucose, TG, HDL cholesterol, and waist circumference, after 8 weeks of RS intervention compared with baseline. Secondary endpoints included changes in gut microbiota composition and inflammatory markers (high-sensitivity C-reactive protein, ferritin (hsCRP), ferritin, and fecal calprotectin) from baseline to week 8. These measures were intended to investigate potential mechanistic links between RS intake, microbial alterations, and host metabolic responses.

### 2.4. Microbiota Analysis

For the analysis of gut microbiota, DNA was isolated from stool samples, and the V3–V4 regions of the 16S rRNA gene were amplified and sequenced using the Illumina MiSeq platform (2 × 250 bp paired-end, Illumina, San Diego, CA, USA). Raw sequence data were processed through established workflows, including adapter trimming with Cutadapt, quality control and chimera removal with DADA2, and amplicon sequence variant (ASV) inference using QIIME2 (v2021.11). Taxonomic classification was conducted using the SILVA database (v138), applying a minimum identity cutoff of 97%. Metrics of alpha diversity (Chao1, Shannon) and beta diversity (Bray–Curtis, UniFrac) were calculated using the Phyloseq R package (version 1.50.0), and statistical differences across groups were tested with the Wilcoxon rank-sum test and PERMANOVA (999 permutations, vegan package (version 2.6.10)). Differentially abundant taxa were determined with LEfSe, with statistical significance assigned to taxa demonstrating an LDA score ≥ 2.0. All statistical analyses were implemented in R (v4.2.0), and graphical representations were produced using ggplot2 (v3.3.5).

### 2.5. Statistical Analysis

The normality of continuous variables was assessed using the Kolmogorov–Smirnov test. All continuous variables, including anthropometric outcomes (e.g., body weight, BMI, waist circumference, skeletal muscle mass, body fat mass, body fat percentage, visceral fat area), dietary intake (total energy, carbohydrates, protein, and fat), and biochemical markers (blood pressure, fasting glucose, liver enzymes, triglycerides, ferritin, hsCRP, and calprotectin), were reported as mean ± standard deviation. The analysis included all enrolled participants (*n* = 30) with no exclusions. Wilcoxon signed-rank tests were used to assess within-group differences from baseline to week 8, while Mann–Whitney U tests were applied for between-group comparisons. A two-sided *p*-value < 0.05 was used to denote statistical significance.

## 3. Results

The nutrient composition of the participants’ habitual diets, based on their dietary records, are shown in Table 2. Both groups showed significant decreases in total caloric consumption (HRS: *p* = 0.031; LRS: *p* = 0.013) and carbohydrate intake (HRS: *p* = 0.021; LRS: *p* = 0.043) when compared to the baseline (Week 0). Reductions in fat and protein intake were more pronounced in the LRS group. Total energy, macronutrient, fiber, and RS intakes before and after the intervention, which included RS and dietary fiber in the participants’ regular diets are also provided in Supplementary Table S1. Total energy, macronutrient, and fiber intake showed no statistically significant changes between baseline and the end of the intervention. Only total RS intake showed a statistically significant increase after the intervention in the HRS group compared with the LRS group (HRS: baseline, 2.6 ± 1.3; 8 weeks, 7.9 ± 1.4; LRS: baseline, 3.1 ± 1.6; 8 weeks, 2.4 ± 1.5; *p* < 0.001).

### 3.1. Primary Outcomes: Metabolic and Anthropometric Parameters

As a primary outcome of RS diet, body composition changes were monitored before and after the 8-week intervention (Table 3). In the HRS group, significant increases were noted in body weight (baseline, 70.3 ± 7.2; 8 weeks, 71.1 ± 7.2; *p* = 0.043), body mass index (BMI) (baseline, 27.8 ± 3.2; 8 weeks, 28.1 ± 3.1; *p* = 0.042), body fat mass (baseline, 27.3 ± 5.5; 8 weeks, 28.3 ± 5.5; *p* = 0.005), body fat percentage (baseline, 38.6 ± 4.2; 8 weeks, 39.7 ± 4.3; *p* = 0.002), and visceral fat area (baseline, 137.4 ± 31.5; 8 weeks, 145.5 ± 33.8; *p* = 0.001). In contrast, no significant changes were detected in these parameters in the LRS group. Skeletal muscle mass remained stable in both groups.

We further monitored changes in metabolic parameters and blood biomarkers (Table 4). Within the HRS group, systolic blood pressure significantly decreased from 133.5 ± 11.5 to 129.3 ± 9.3 mmHg (*p* = 0.010), while serum TG concentrations increased from 127.5 ± 47.1 to 168.7 ± 93.5 mg/dL (*p* = 0.017). The LRS group, however, exhibited no significant changes in these parameters. Glucose and liver enzyme levels remained largely unchanged, except for a minor reduction in AST within the LRS group (*p* = 0.049).

### 3.2. Secondary Outcomes: Inflammatory Markers and Gut Microbiota

We furthermore monitored inflammatory biomarkers—ferritin, hsCRP, and fecal calprotectin—that are best-studied non-invasive biomarkers in inflammatory bowel disease. These biomarkers did not exhibit significant changes between or within groups after the intervention period (Table 4). Baseline hsCRP levels were lower in the HRS group than in the LRS group, but this difference disappeared after 8 weeks of diet.

**Figure 1:**
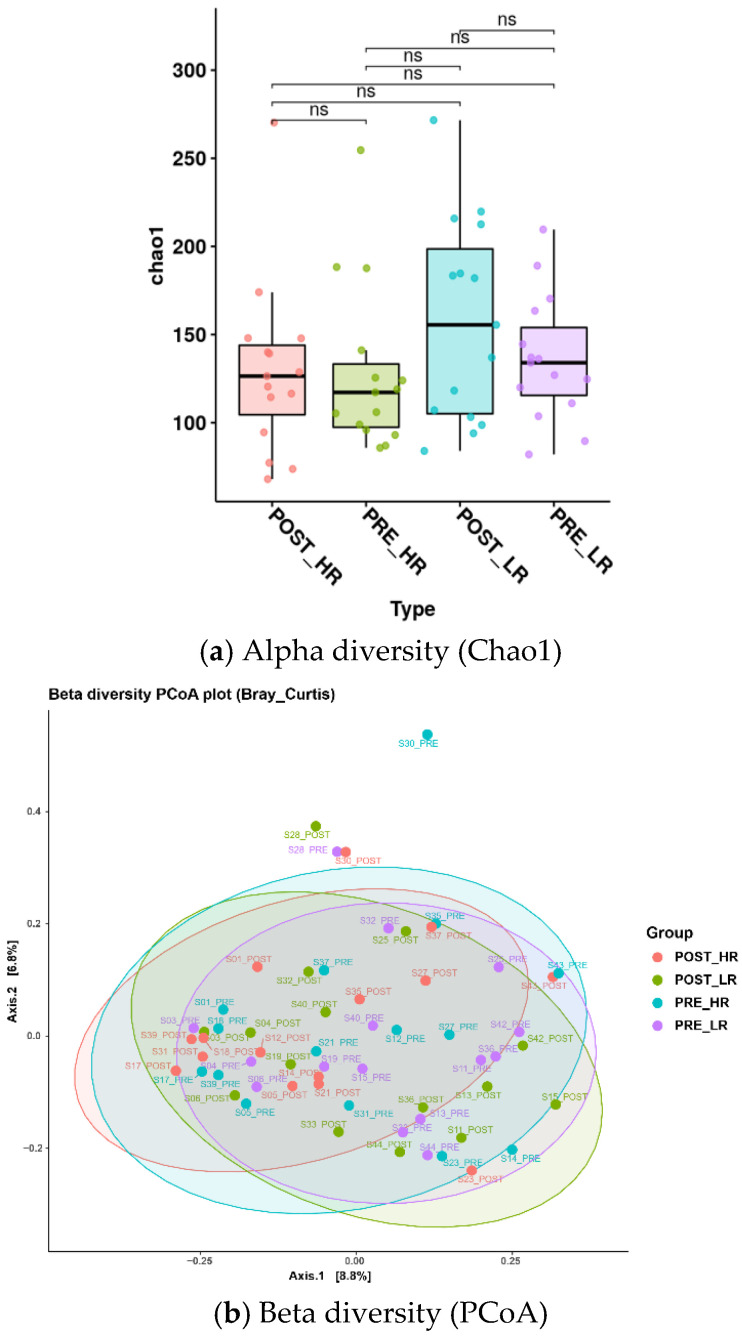
Comparison of alpha diversity (**a**) and beta diversity (**b**) in high-resistant and low-resistant starch groups before and after the intervention. The Chao1 index for alpha diversity indicated no statistically significant differences either within groups or between groups pre- and post-intervention, as assessed using the Wilcoxon rank-sum test. Similarly, beta diversity (PCoA) showed no significant variation attributable to the intervention (*p* > 0.05). POST_HR, post-intervention_high-resistant; PRE_HR, pre-intervention_high-resistant; POST_LR, post-intervention_low-resistant starch; PRE_LR, pre-intervention_low-resistant starch. ns, not significant.

To assess the impact of RS on gut microbiota composition and diversity, we profiled microbial library of stools from individuals with HRS and LRS. Figure 1 illustrates alpha and beta diversity indices of the gut microbiota. The Chao1 index (alpha diversity) showed no significant intra- or inter-group differences before and after the intervention (Figure 1a), as determined by the Wilcoxon rank-sum test. Beta-diversity analyses based on PCoA indicated no overall community-level shifts in microbiomes before and after diet (*p* > 0.05) (Figure 1b).

**Figure 2:**
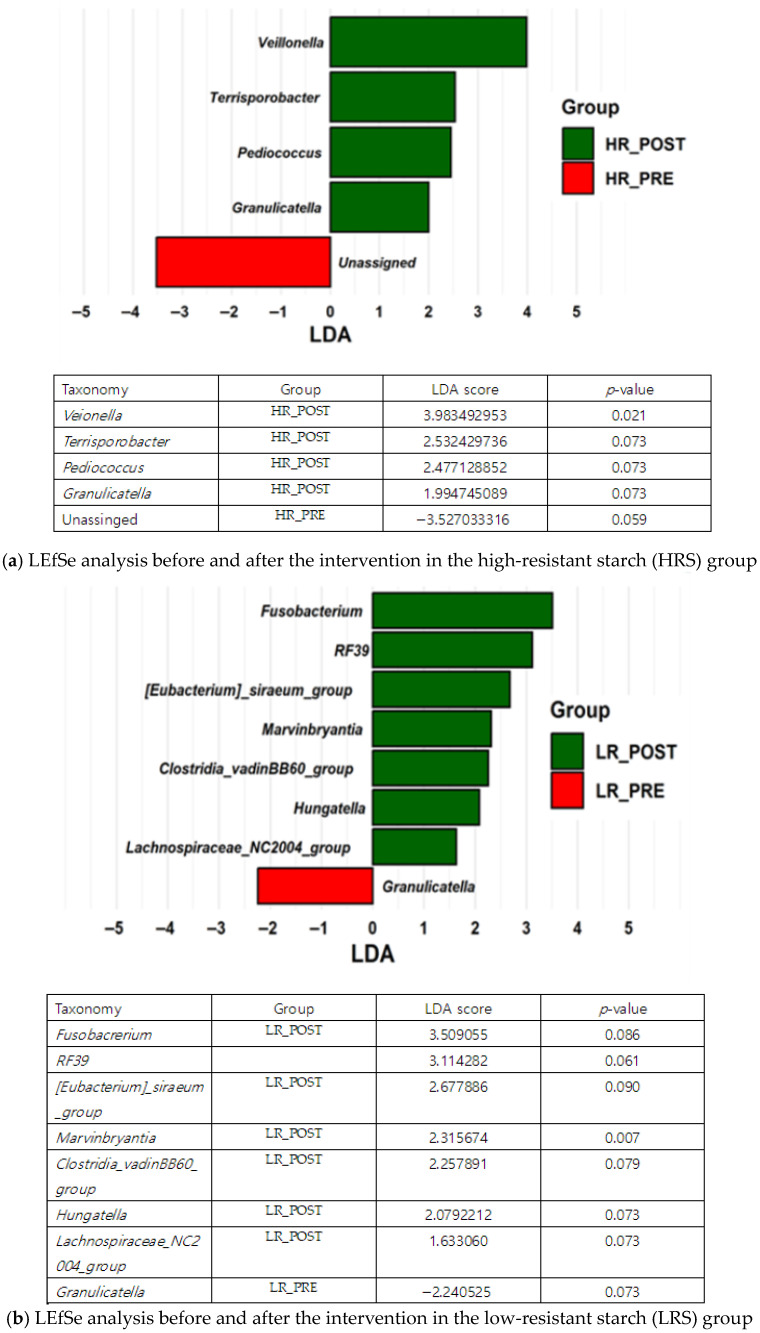
Linear Discriminant Analysis Effect Size (LEfSe) (high-resistant, low-resistant Group, Pre vs. Post). Only features with an LDA score greater than 1.0 are shown. (**a**) After the 8 weeks of diet, a significant increase in *Veillonella* abundance was found in the HRS group. (**b**) The LRS group exhibited a marked increase in *Marvinbryantia* following the intervention. HR_POST, post-intervention_high-resistant; HR_PRE, pre-intervention_high-resistant; LR_POST, post-intervention_low-resistant starch; LR_PRE, pre-intervention_low-RS.

We further identified different taxa by Linear Discriminant Analysis Effect Size (LEfSe). The genus *Veillonella* significantly increased in the HRS group after diet, whereas *Marvinbryantia* showed a significant enrichment in the LRS group (Figure 2a,b).

## 4. Discussion

This prospective study examined the dose-dependent effects of RS intake on metabolic parameters, body composition, and gut microbiota among women exhibiting metabolic syndrome risk factors. Participants were allocated into two groups: the HRS group, which received cereal and energy bars enriched with RS, and the LRS group, which received comparable products containing a low amount of RS. The findings revealed that the HRS group experienced significant increases in body fat and TG levels, as well as a modest decrease in systolic blood pressure than the LRS group.

The observed increase in adiposity and visceral fat within the HRS group is unexpected, considering the well-documented health benefits of dietary fiber and RS. Nevertheless, this outcome could be linked to modifications in the gut microbiota resulting from RS fermentation [2]. This study specifically discovered altered microbiomes by RS, indicating a significant rise in the genus *Veillonella* in the HRS group. *Veillonella* is recognized for its ability to metabolize lactate into SCFAs [20], which may provide additional energy substrates that contribute to increased body weight [21]. Moreover, the increase in body fat, despite the absence of any change in total caloric intake before and after the intervention, may reflect enhanced colonic fermentation and SCFA-mediated energy recovery from undigested carbohydrates, consistent with previous findings that *Veillonella* converts lactate into acetate and propionate, thereby promoting hepatic lipogenesis [20,22]. On the other hand, the LRS group exhibited significantly decreased fat and protein intake 8 weeks after the intervention compared to baseline (week 0), but showed no significant alterations in body weight or composition.

Notably, while the HRS group demonstrated a statistically significant decrease in systolic blood pressure, this occurred alongside a marked elevation in TG levels (mean change ≈ +40 mg/dL). This dual effect highlights the intricate and multifactorial metabolic consequences of RS. The decline in blood pressure may be attributable to enhanced production of gut-derived SCFAs and potentially associated anti-inflammatory actions [23,24,25]. Conversely, the increase in TG could result from augmented hepatic lipogenesis driven by elevated SCFA absorption [22]. Therefore, given the clinically meaningful rise in TG levels, dietary interventions involving HRS should include regular lipid monitoring to ensure cardiometabolic safety.

The analysis of inflammatory markers indicated only subtle changes. At baseline, HsCRP levels were significantly lower in the HRS group compared to the LRS group, but this difference was no longer apparent after the intervention. The concentrations of serum ferritin, a marker for subclinical inflammation in blood [26], and calprotectin, which identifies micro-inflammation in the colon [27], did not significantly differ between the diet groups at any time point. It is plausible that the relatively short duration of the intervention was insufficient to produce measurable reductions in serum ferritin or fecal calprotectin, as these biomarkers may require a longer exposure period to dietary modulation for significant change. This interpretation is consistent with a recent meta-analysis [28] supporting that RS intake reduced interleukin-6 levels without affecting CRP concentrations. Taken together, these findings emphasize the need for longer-term studies to clarify the anti-inflammatory regulatory roles of RS.

Microbial profiling also revealed distinct compositional responses to RS supplementation between the two groups. The HRS group exhibited an increased abundance of *Veillonella*, a genus associated with lactate utilization and propionate/acetate production, whereas the LRS group showed higher levels of *Marvinbryantia*, a butyrate-producing member of the Lachnospiraceae family [29]. These contrasting microbial alterations suggest distinct metabolic routes of RS and downstream consequences.

*Veillonella* species convert lactate into propionate and acetate via the methylmalonyl-CoA pathway [30], and the byproduct SCFAs can serve as additional energy substrates for hepatic metabolism. Acetate contributes to *de novo* lipogenesis, whereas propionate may promote both gluconeogenic and lipogenic pathways by activating the Sterol Regulatory Element-Binding Protein 1 (SREBP-1), which subsequently enhances the expression of lipogenic genes [31]. Altogether, these findings provide a plausible mechanistic explanation for the elevated adiposity and TG levels observed in the HRS group [32]. Conversely, *Marvinbryantia* primarily produces butyrate that serves as the main energy source for colonocytes and exerts anti-inflammatory signaling and lipid-lowering effects, through AMP-activated protein kinase activation and histone deacetylase inhibition [33]. This metabolic distinction aligns with the relatively stable lipid indices seen in the LRS group and suggests that the predominant SCFA type—acetate/propionate versus butyrate—may differentially influence host lipid homeostasis.

Consistent with these mechanistic interpretations, the HRS-fed group exhibited significant alterations in several metabolic outcomes with a concurrent increase in *Veillonella* at the genus level, while the LRS-fed group had only modest metabolic shifts accompanied by a rise in *Marvinbryantia*. These findings collectively suggest that *Veillonella* may exert a stronger modulatory influence on host metabolic functions compared to *Marvinbryantia*.

To place these findings in context, we assessed participants’ habitual intake of RS and dietary fiber. Based on previous reports describing the average daily consumption of these nutrients among Korean adults, the intakes of RS and dietary fiber in both the LRS and HRS groups were comparable to typical Korean dietary levels (mean adult RS intake ≈ 3 g/day, range 1–7 g/day; mean adult fiber intake ≈ 22 g/day, range 19–27 g/day) [14,34]. This suggests that the LRS group reflects the habitual range of RS consumption commonly observed in the Korean population.

We observed no detectable shifts in alpha or beta diversity over the 8-week intervention period; however, this finding should be interpreted with caution. Microbial diversity indices often require longer intervention durations or more substantial dietary perturbations to demonstrate measurable changes. In addition, substantial inter-individual variability and differences in baseline microbiota compositions may obscure subtle community-level shifts, even when specific taxa (e.g., *Veillonella* and *Marvinbryantia*) exhibit significant alterations. Given the modest sample size and relatively short intervention period, the study may have been underpowered to detect diversity-level changes.

Our study has notable strengths. This present study utilizes a randomized prospective design, which reduces the risk of selection bias and improves the reliability of comparisons between the HRS and LRS groups. Second, the intervention included real food items, such as cereals and energy bars, thereby enhancing the applicability of the findings to everyday dietary scenarios rather than simulated supplementation alone. Third, a comprehensive range of measures was assessed, encompassing anthropometrics, metabolic and inflammatory markers, as well as detailed gut microbiota analyses, thus providing an integrated assessment of functional role of RS. Fourth, the study addresses a critical population, by focusing on women at increased risk for metabolic disorders, raising the practical nutritional significance of the results. Nonetheless, our study has limitations; First, the intervention duration was relatively short, which may have constrained the ability to observe long-term metabolic effects of HRS and LRS. Second, the small sample size, without a formal power calculation, limits the statistical power of the findings. Third, the reliance on self-reported dietary intake and physical activity data may have introduced recall bias, with limited objective adherence checks. Fourth, differences in participants’ baseline microbiota composition could have influenced individual metabolic responses to RS. Fifth, the female-only cohort limits the generalizability of the findings to broader populations. Sixth, the short follow-up period may not have been sufficient to capture microbiome remodeling, and the possible influence of baseline microbial heterogeneity on metabolic responses cannot be ruled out. Finally, the analysis included multiple endpoints without adjustment, which may increase the risk of type I error. Future research should adopt personalized nutrition approaches and include extended follow-up periods to identify the most effective types and dosages of RS for optimizing metabolic health and capturing long-term outcomes.

## 5. Conclusions

In conclusion, supplementation with RS in women presenting risk factors for metabolic health yielded complex metabolic outcomes. From a clinical perspective, these findings suggest that clinicians and dietitians should carefully monitor TG levels and body composition when recommending RS supplementation, particularly in individuals with metabolic risk factors. Tailoring the type and dosage of RS to each patient’s metabolic profile may help maximize cardiovascular benefits while minimizing potential adverse effects on adiposity. Overall, these results underscore the importance of individualized dietary interventions and highlight the need for further mechanistic studies to elucidate the interactions among RS, gut microbiota, and host metabolic pathways.

## Figures and Tables

**Table 1:** Composition of nutrients and resistant starch content in low-resistant starch (LRS) (a) and high-resistant starch (HRS) (b) groups per serving provided.

	Nutrient Composition	Food Item	Serving Size (g)	Energy (kcal)	Carbohydrates (g)	Protein (g)	Fat (g)	Dietary Fiber (g)	Resistant Starch (g)
(a) LRS Group		Mixed grain powder	40	157.79	31.54	5.20	1.81	2.75	0.06
		Breakfast cereal	50	219.12	33.38	8.20	8.20	3.22	0.09
		Energy bar	95	288.14	42.20	10.10	10.31	6.92	0.31
	Total	—	185	665.05	107.12	19.87	20.32	12.88	0.47
(b) HRS Group		Mixed grain powder	40	150.73	32.39	4.64	0.91	2.80	3.90
		Breakfast cereal	50	212.42	29.98	5.06	9.57	6.95	1.24
		Energy bar	100	274.82	46.42	9.51	7.42	7.80	2.54
	Total	—	190	637.97	108.79	19.21	17.90	17.55	7.67

**Table 2:** Pre- and Post-Intervention Changes in Dietary Intake.

Variable	HRS (Week 0)	HRS (Week 8)	*p*-Value	LRS (Week 0)	LRS (Week 8)	*p*-Value
Total Calories (kcal)	1749.5 ± 483.2	1345.1 ± 624.4	0.031 *	1664.5 ± 370.8	1255.4 ± 404.7	0.013 *
Carbohydrates (g)	248.7 ± 76.6	186.7 ± 78.2	0.021 *	230.2 ± 44.3	181.3 ± 63.1	0.043 *
Fat (g)	49.1 ± 15.2	39.5 ± 23.1	0.170	50.3 ± 19.7	35.0 ± 14.5	0.007 **
Protein (g)	71.4 ± 28.0	62.0 ± 38.5	0.431	66.6 ± 17.6	49.8 ± 19.0	0.015 *

Data are shown as mean ± standard deviation, HRS, high-resistant starch; LRS, low-resistant starch; *p*-value calculated by Wilcoxon signed-rank test. * indicates *p* < 0.05, ** *p* < 0.01.

**Table 3:** Pre- and Post-Intervention Changes in Body Composition.

Variable	HRS (Week 0)	HRS (Week 8)	*p*-Value	LRS (Week 0)	LRS (Week 8)	*p*-Value
Weight (kg)	70.3 ± 7.2	71.1 ± 7.2	0.043 *	72.8 ± 6.3	73.0 ± 6.0	0.601
Waist Circumference (cm)	91.8 ± 5.5	92.7 ± 5.4	0.115	94.1 ± 8.2	93.9 ± 8.8	0.740
BMI (kg/m^2^)	27.8 ± 3.2	28.1 ± 3.1	0.042 *	28.4 ± 2.8	28.5 ± 2.7	0.580
Skeletal Muscle Mass (kg)	23.4 ± 2.0	23.2 ± 2.0	0.194	24.2 ± 2.3	24.0 ± 2.2	0.399
Body Fat Mass (kg)	27.3 ± 5.5	28.3 ± 5.5	0.005 **	28.4 ± 5.6	28.9 ± 5.5	0.101
Body Fat Percentage (%)	38.6 ± 4.2	39.7 ± 4.3	0.002 **	38.7 ± 5.6	39.4 ± 5.4	0.069
Visceral Fat Area (cm^2^)	137.4 ± 31.5	145.5 ± 33.8	0.001 **	135.7 ± 35.4	145.0 ± 36.3	0.089

Values are presented as mean ± standard deviation; HRS, high-resistant starch; LRS, low-resistant starch; BMI, body mass index; *p*-value determined by Wilcoxon signed-rank test; * indicates *p* < 0.05, ** *p* < 0.01.

**Table 4:** Pre- and Post-Intervention Changes in Metabolic and Inflammatory Parameters.

**Variable**	**HRS (Week 0)**	**HRS (Week 8)**	***p*-Value ^a^**	**LRS (Week 0)**	**LRS (Week 8)**	***p*-Value ^a^**
Systolic BP (mmHg)	133.5 ± 11.5	129.3 ± 9.3	0.010 *	129.2 ± 12.6	127.9 ± 12.0	0.525
Glucose (mg/dL)	92.3 ± 6.3	96.9 ± 9.5	0.094	102.1 ± 11.8	103.7 ± 9.2	0.295
AST (mg/dL)	30.0 ± 19.8	23.2 ± 9.7	0.075	26.3 ± 12.0	21.5 ± 7.9	0.049 *
TG (mg/dL)	127.5 ± 47.1	168.7 ± 93.5	0.017 *	143.0 ± 57.8	158.7 ± 59.0	0.348
	**HRS (Week 0)**	**LRS (Week 0)**	***p*-Value ^b^**	**HRS (Week 8)**	**LRS (Week 8)**	***p*-Value ^b^**
Ferritin (ug/L)	135.5 ± 151.0	104.9 ± 117.2	0.305	129.9 ± 131.8	91.7 ± 87.1	0.436
hsCRP (mg/dL)	0.10 ± 0.07	0.19 ± 0.15	0.037 *	0.11 ± 0.07	0.13 ± 0.10	0.806
Calprotectin (mg/kg)	39.9 ± 62.3	48.7 ± 72.4	0.744	34.8 ± 78.2	45.1 ± 61.1	0.305

Results presented as mean ± standard deviation; HRS, high-resistant starch; LRS, low-resistant starch; BP, Blood pressure; AST, Aspartate aminotransferase; TG, triglycerides; hsCRP, High-sensitivity C-reactive protein; ^a^: *p*-value by Wilcoxon signed-rank test, ^b^: *p*-value by Mann–Whitney U test. * indicates *p* < 0.05.

## Data Availability

The original contributions presented in this study are included in the article and Supplementary Materials. Further inquiries can be directed to the corresponding author.

## Supplementary Materials

The following supporting information can be downloaded at: https://www.mdpi.com/article/10.3390/nu17233652/s1, Table S1: Dietary intake of macronutrients, dietary fiber, and total resistant starch in the HRS and LRS groups (baseline and 8 weeks).
