# Supplementary material for: Effects of Resistant Starch on Metabolic Markers and Gut Microbiota in Women with Metabolic Syndrome Risk Factors: A Randomized, Double-Blind, Pilot Study
Source: Nutrients. 2025 Nov 21;17(23):3652. doi: 10.3390/nu17233652 (PMC12693806; doi:10.3390/nu17233652)
Supplement: Supplementary file 1 [file nutrients-17-03652-s001.zip › nutrients-3988463-supplementary.pdf]

Supplementary Table

Table S1. Dietary intake of macronutrients, dietary fiber, and total resistant starch in the HRS and LRS groups (baseline and 8 weeks).

Variable	HRS Week 0	HRS Week 8	LRS Week 0	LRS Week 8	p-value*
Energy (kcal)	1714.5 ± 384.5	1796.2 ± 420.5	1664.8 ± 390.6	1755.8 ± 401.6	0.965
Carbohydrate(g)	244.4 ± 43.1	270.1 ± 55	230.5 ± 53	259.2 ± 64.2	0.922
Protein(g)	70.4 ± 26.8	70.3 ± 25.6	66.9 ± 20.8	68.7 ± 20.3	0.836
Fat(g)	47.9 ± 19.5	49.1 ± 19	50.5 ± 22.1	50.5 ± 14.4	0.870
Dietary fiber (g)	19.3 ± 5.7	26.6 ± 6.1	16.5 ± 4.9	25.5 ± 4.3	0.495
Total resistant starch (g)	2.6 ± 1.3	7.9 ± 1.4	3.1 ± 1.6	2.4 ± 1.5	<0.001

* To compare the changes between the HRS and LRS groups before and after the intervention, the Mann–Whitney U test was performed. HRS, high-resistant starch; LRS, low-resistant starch
